# HSCs-derived COMP drives hepatocellular carcinoma progression by activating MEK/ERK and PI3K/AKT signaling pathways

**DOI:** 10.1186/s13046-018-0908-y

**Published:** 2018-09-19

**Authors:** Qing Li, Cong Wang, Yufeng Wang, Liankang Sun, Zhikui Liu, Liang Wang, Tao Song, Yingmin Yao, Qingguang Liu, Kangsheng Tu

**Affiliations:** Department of Hepatobiliary Surgery, the First Affiliated Hospital of Xi’an Jiaotong University, 277 Yanta West Road, Xi’an, 710061 Shaanxi Province China

**Keywords:** Hepatocellular carcinoma, Microenvironment, Hepatic stellate cells, COMP, Tumor progression

## Abstract

**Background:**

Cartilage oligomeric matrix protein (COMP) is known to promote fibrosis in skin, lung and liver. Emerging evidence shows that COMP plays critical roles in tumor development, including breast cancer, colon cancer and hepatocellular carcinoma (HCC). Nevertheless, the role of COMP in HCC proliferation and metastasis and its underlying mechanisms remain fully unclear.

**Methods:**

Serum COMP was determined by ELISA. Cell Counting Kit-8 and plate colony formation were performed to evaluate cell proliferation. Wound healing and transwell assays were used to determine migration and invasion of HCC cells. Western blotting and immunofluorescence were carried out for detection of epithelial-to-mesenchymal transition (EMT) markers and MMPs in HCC cells. The in vivo role of COMP was evaluated using mouse models. We also measured effects of hepatic stellate cells (HSCs)-conditioned medium (CM) on HCC progression using transwell coculture system.

**Results:**

Here, we found that serum COMP levels in HCC patients were significantly higher than those in healthy controls. Accordingly, high serum COMP levels in HCC patients significantly correlated with malignant clinical characteristics and poor clinical outcomes. Next, we investigated that recombinant human COMP protein (rCOMP) treatment resulted in increased abilities of proliferation, invasion and migration of HCC cells. Furthermore, rCOMP treatment enhanced proliferative and metastatic colonization of HCC cells in vivo. Mechanistically, CD36 receptor played an essential role in COMP-mediated HCC cell proliferation and metastasis. Functionally, COMP/CD36 signaling caused phosphorylation of ERK and AKT, resulting in the upregulation of tumor-progressive genes such as EMT markers, MMP-2/9, Slug and Twist in HCC cells. Interestingly, we revealed that COMP was secreted by HSCs. CM of LX2 cells with COMP knockdown showed weaker effects on the activation of MEK/ERK and PI3K/AKT signaling pathways in HCC cells compared to control CM.

**Conclusions:**

Our findings indicated that HSCs-derived COMP collaborated with CD36 and subsequently played an essential role in MEK/ERK and PI3K/AKT-mediated HCC progression. COMP might act as a promising target for the diagnosis and treatment of aggressive HCC.

**Electronic supplementary material:**

The online version of this article (10.1186/s13046-018-0908-y) contains supplementary material, which is available to authorized users.

## Background

Hepatocellular carcinoma (HCC) is the most common malignancies worldwide with a third rank of mortality rate in all types of cancer [1]. HBV or HCV, exposure to alcohol intoxication are major risk factors which induced hepatic inflammation and fibrosis and consequently lead to cirrhosis as observed in 80% of HCC patients [2]. Elucidating the molecular signaling mechanisms of cirrhosis to HCC and the search for the novel therapeutic targets and treatment are of great significance in improving the overall prognosis of HCC patients.

Cartilage oligomeric matrix protein (COMP), a cartilage metabolism marker, is an extracellular matrix protein that modulates the cellular phenotype during tissue genesis and remodeling. Recently, it has been extensively studied for its pro-fibrosis potential against various internal organs, for instance, pulmonary fibrosis [3], and liver cirrhosis [4]. Earlier work has suggested that COMP levels in the serum of HCC patients and HCC tissues are abnormally elevated compared with healthy controls and it can be used as diagnostically in the non-invasive estimation of liver cirrhosis and HCC development [5, 6]. Recent studies have shown that COMP promotes the progression of breast cancer, colon cancer and prostate cancer [7–9]. These results suggest that COMP may be an important pro-HCC molecule, but the mechanism by which COMP plays a role in HCC still needs to be further studied.

CD36 is a traditional membrane receptor of the thrombospondin family that binds to CD36 thereby activating downstream signaling pathways involved in various cellular processes. In HCC, CD36 is abnormally high which promotes EMT process by increasing free fatty acid uptake [10, 11]. Increasing evidences indicate that EMT contributes to tumor metastasis and chemotherapy resistance of HCC [12–16]. Transcriptional repressors of epithelial gene such as Snail, Slug and Twist are essentially involved in EMT and either directly or indirectly induced by signaling from MEK/ERK or PI3K/AKT [17]. Recent studies have found that COMP binds to CD36 and activates downstream signaling pathways and ultimately leading to the progression of liver cirrhosis [4, 18].

In the present study, we specifically investigated the effect of COMP on the malignant behaviors of HCC by in vivo and in vitro experiments, including proliferation, migration and invasion. Notably, we discovered that activated hepatic stellate cells (HSCs)-derived COMP regulated mesenchymal gene expression and MMPs in HCC cells via CD36 and caused aberrant phosphorylation of ERK and AKT. Analysis of primary HCC serum samples supported the predictive role of COMP in patient’s survival, suggesting that COMP was a promising biomarker and an effective bioactive strategy to combat tumor-progression.

## Methods

### Cell culture and treatment

The HCC cell lines (MHCC-97H, HepG2, Huh7, Hep-3B, SMMC-7721) and the immortalized human liver cell line LO2 and activated hepatic stellate cells (HSCs) LX2 were purchased from the Shanghai cell bank (Shanghai Institute for Biological Science, Chinese Academy of Science, Shanghai, China) and cultured at 37 °C in a humidified atmosphere with 5% CO_2_ in high-glucose Dulbecco’s Modified Eagle’s Medium (DMEM; Invitrogen, NY, USA) supplemented with 10% fetal bovine serum (FBS; Hyclone, Logan, UT, USA) and 1% penicillin-streptomycin. The culture medium was replaced with serum-free DMEM 12 h before treatment with 0–5 μg/ml of human rCOMP (R&D Systems, Minneapolis, MN) according to the manufacture’s recommendations.

### Clinical samples and ELISA

Venous blood samples of 100 HCC patients without other complications (before the surgery) and 30 healthy volunteers were obtained from the First Affiliated Hospital of Xi’an Jiaotong University between 2015 and 2017, the serum was obtained after centrifugation at 3, 000 rpm for 15 min and stored at − 80 °C. Ethic permission was obtained from the Ethics Committee of the First Affiliated Hospital of Xi’an Jiaotong University. Written informed consent was obtained from each patient or family members.

Quantitative measurement of human COMP in serum of HCC patients were performed by human COMP ELISA kit (R&D System, Minneapolis, MN) following the manufacturer’s protocols. Briefly, serum of HCC patients was collected before the surgery, cell supernatants were collected after cultivated in serum-free medium for 24 h. A seven-point standard curve was generated for every plate and quantified using the GraphPad Prism 5.0 software (La Jolla, USA). All samples were analyzed in triplicates.

### In vitro cell proliferation and plate colony-forming assays

The cell proliferation was measured using the Cell Counting Kit-8 (CCK-8) (Dojindo, Kyushu, Japan). Cells were seeded in 96-well plates at 4 *×* 10^3^ cells/well and incubated overnight to allow their adhesion to the plate. Cells were treated with rCOMP in different concentrations (0, 0.8, 1, 2 and 5 μg/ml) for 12 h, 24 h, and 36 h, five parallel wells for each concentration. Ten microliters of CCK8 (Sigma-Aldrich, St. Louis, MO, USA) was added to 90ul of the medium per well and incubated at 37 °C for 4 h. The absorbance was detected at 450 nm with microplate autoreader (Bio-Rad, CA, USA).

For the colony formation assay, Hep-3B and SMMC-7721 cells in exponential growth phase were seeded into 6-well plates at a density of 1000 cells/well. After overnight incubation, cells were treated with rCOMP (0, 1, 2 and 5 μg/ml) and maintained in culture medium for 2 weeks and the medium with corresponding rCOMP concentrations was replaced every three days. The colonies were fixed with 4% methanol and stained with 0.1% crystal violet at room temperature and the number of colonies was counted. Data were collected from three independent experiments.

### Wound-healing assay and Transwell migration and invasion assays

The cells were grown to a 90–100% confluence cell monolayer overnight prior to serum starvation for 8 h in 6-well plates. After wounding with a sterile pipette tip, cells were then washed with PBS to eliminate the floating cells. Cells were treated with rCOMP (0, 1, 2 and 5 μg/ml) and the wounds were photographed at time 0 h and 24 h post-wounding under a phase-contrast microscope. Cell migration was quantitated by measuring the width of the wounds. Migration rate was calculated as (%) = [W (24 h) - W (0 h)]/W (0 h). Experiments were performed with at least three times.

The migration assay was performed in a Transwell chemotaxis 24-well chamber (BD Biosciences, Franklin Lakes, NJ), 2 × 10^4^ cells in 200 μl serum-free medium were plated in the upper chambers. For invasion assay, the basement membrane of filters was coated with 50 μl Matrigel (Matrigel; BD Biosciences, Bedford, MA). After incubation with rCOMP (0, 1, 2 and 5 μ g/ml) for 24 h, cells migrated or invaded to the lower surface of the membrane were stained with crystal violet. The result was determined by counting the stained cells using optical microscopy (200 × magnifications) in five randomly selected fields. Each experiment was carried out in triplicate wells and repeated at least three times.

### Western blot analysis

Western blot analysis was performed to detected the levels of COMP (ab11056, Abcam, Cambridge, UK), CD36 (ab133625, Abcam), E-cadherin (3195, Cell Signaling Technology, Danvers, USA), N-cadherin (14,215, Cell Signaling Technology), Vimentin (5741, Cell Signaling Technology), MMP-2 (13,132, Cell Signaling Technology), MMP-9 (sc-393,859, SANTA CRUZ), Snail (ab167609, Abcam), Slug (9585, Cell Signaling Technology), Twist (ab175430, Abcam), AKT (4691, Cell Signaling Technology), P-AKT (Thr308) (13,038, Cell Signaling Technology), ERK (5013, Cell Signaling Technology), P-ERK (4370, Cell Signaling Technology), ki-67 (ab15580, Abcam), α-SMA (ab5694, Abcam), β-actin (sc-47,778, SANTA CRUZ). Cells treated with rCOMP (0, 1, 2 and 5 μg/ml) were planted in 6-well plates for 24 h or 48 h, and lysed in lysis buffer (Invitrogen). Protein concentration was determined by the BCA Kit (Pierce, IL, USA) and 40 μg of each samples was separated by sodium dodecyl sulfate–polyacrylamide gel electrophoresis (SDS-PAGE) and transferred to polyvinylidene difluoride (PVDF) membranes (Millipore, Bedford, MA, USA). After incubated with primary antibodies overnight at 4 °C, the membranes were then incubated with the appropriate HRP-conjugated secondary antibody for 1 h at room temperature and visualized using the Bio-Rad Gel imaging system and analyzed by the software program as specified by Bio-Rad.

### Immunohistochemical analysis (IHC) and immunofluorescence (IF) analysis

The tumor tissue sections embedded in paraffin were incubated with ki-67 (1:200), CD36 (1:200), CD36 (1:200), E-cadherin (1:200), N-cadherin (1:200) and Vimentin (1:200) antibodies.

For immunofluorescence staining, treated cells were stained with E-cadherin (1:100; Cell Signaling Technology), Vimentin (1:100; Cell Signaling Technology) overnight at 4 °C, followed by incubation with corresponding FITC-conjugated secondary antibody (Invitrogen) for 1 h at room temperature. Cells were quantified by confocal immunofluorescence microscopy (Zeiss, Oberkochen, Germany).

### Cell transfections

For CD36 stable knockdown assay, lentiviral containing short hairpin RNAs specially targeting CD36 (shCD36, sense: 5’-GUACCCUGUUACUACCACAdTdT-3′, antisense: 5’-UGUGGUAGUAACAGGGUACdTdT-3′) and the scramble control short hairpin RNA (shCtl) cloned were purchased from GeneChem Corporation (Shanghai, China) and transfected into SMMC-7721 cells using Lipofectamine 2000 according to the manufacturer’s instructions. Experiments were conducted 48 h and knockdown efficiency was verified by Western blot.

For COMP knockdown assay in LX2 cells, small interfering RNA (siRNA) specific to COMP (siRNA1: sense: 5’-AGAAACUUGAGCUGUUGAUGCC-3′, antisense: 5’-GGCUAUCAAGACAGCUCAAGUUUCU-3′; siRNA2: sense: 5’-GAGACAAGATCGACGTGTGTC-3′, antisense: 5’-GACACACGTCGATCTTGTCTC-3′) and the scramble siRNA (NC siRNA) were purchased from Biomics Biotechnologies (Guangzhou, PR China). The cells were plated into 6-well plates and then transfected with 100 nM siRNA using Lipofectamine 2000 (Invitrogen, Eugene, OR, USA) according to the manufacturer’s instructions. Cells were collected for further investigation at the indicated hours after transfection.

### Animal experiments

All animal experiments were conducted in compliance with ethical regulations and approved by the ethical committee of animal care of the Xi’an Jiaotong University, Xi’an, China. For the in vivo tumor formation, 10 female BALB/C nude mice aged 4 weeks (Shanghai SLAC Laboratory Animal Center of Chinese Academy of Sciences, Shanghai, China) were used to establish subcutaneous tumor model. 1 *×* 10^6^ SMMC-7721 cells, which were used in our in vitro experiments, were suspended in 100 μl PBS and incubated with rCOMP (2 μg/ml) at 37 °C for 2 h prior to injection. Then SMMC-7721/rCOMP and SMMC-7721/PBS were subcutaneously injected into the left and right (control) flanks of mice. Tumor growth was monitored by estimating the tumor volume by the formula “a/2 *×* b^2^”, in which a and b represents the largest and smallest diameters, respectively. The mice were sacrificed at day 7. This animal model was applied in our previous publication [19].

For the effect of COMP on pulmonary metastasis was examined by intravenous tail veil injections experiment of 1 × 10^6^ SMMC-7721 cells with or without 2 h rCOMP pre-incubation. In addition, experimental animals (*n* = 6/group) received either rCOMP (1 mg/kg/d) or PBS 3 times per week by tail vein injections. The dose was calculated based on the cutoff value of COMP level in human [20]. The mice were sacrificed 4 weeks and the lung metastases were confirmed by H&E staining.

For assessment of the function of CD36 in vivo, an orthotopic liver tumor model in nude mice was established. Briefly, 1 *×* 10^6^ SMMC-7721-shCD36 and SMMC-7721-shCtl cells (*n* = 5/group) were suspended in 100ul PBS and incubated with rCOMP for 2 h subcutaneously injected into nude mice liver as described [21]. Subsequently, these mice were injected with rCOMP at doses of 1 mg/kg/d or PBS through tail vein, 3 times per week for the duration of the experiment. After 4 weeks the livers and lungs were collected and prepared for further analysis.

### Transwell coculture system

Hep-3B or SMMC-7721/LX2 cocultures were conducted in serum-free DMEM for 24 h using transwell inserts (6.5 mm diameter with polycarbonate membrane filters containing 0.4 μM pores, Corning, NY) which allow diffusion of media components but prevent cell migration (Corning Inc., NY). HCC cells were collected for further investigation.

### Statistical analysis

All the data were expressed as mean ± standard deviation (SD) of 3 independent experiments. Prism 5 and SPSS 13.0 software were applied for all statistical analyses. Pearson chi-square test, ANOVA and Student’s t-test were used for comparison between multiple or two groups. Kaplan-Meier method with Log-rank test were used for survival analysis. Univariable and multivariable survival analyses were performed by Cox proportional hazards regression model. *P < 0.05 and **P < 0.01 were taken as indicative of statistically significant difference.

## Results

### Increased serum level of COMP is associated with poor clinicopathological features and clinical outcomes of HCC patients

**Fig. 1:**
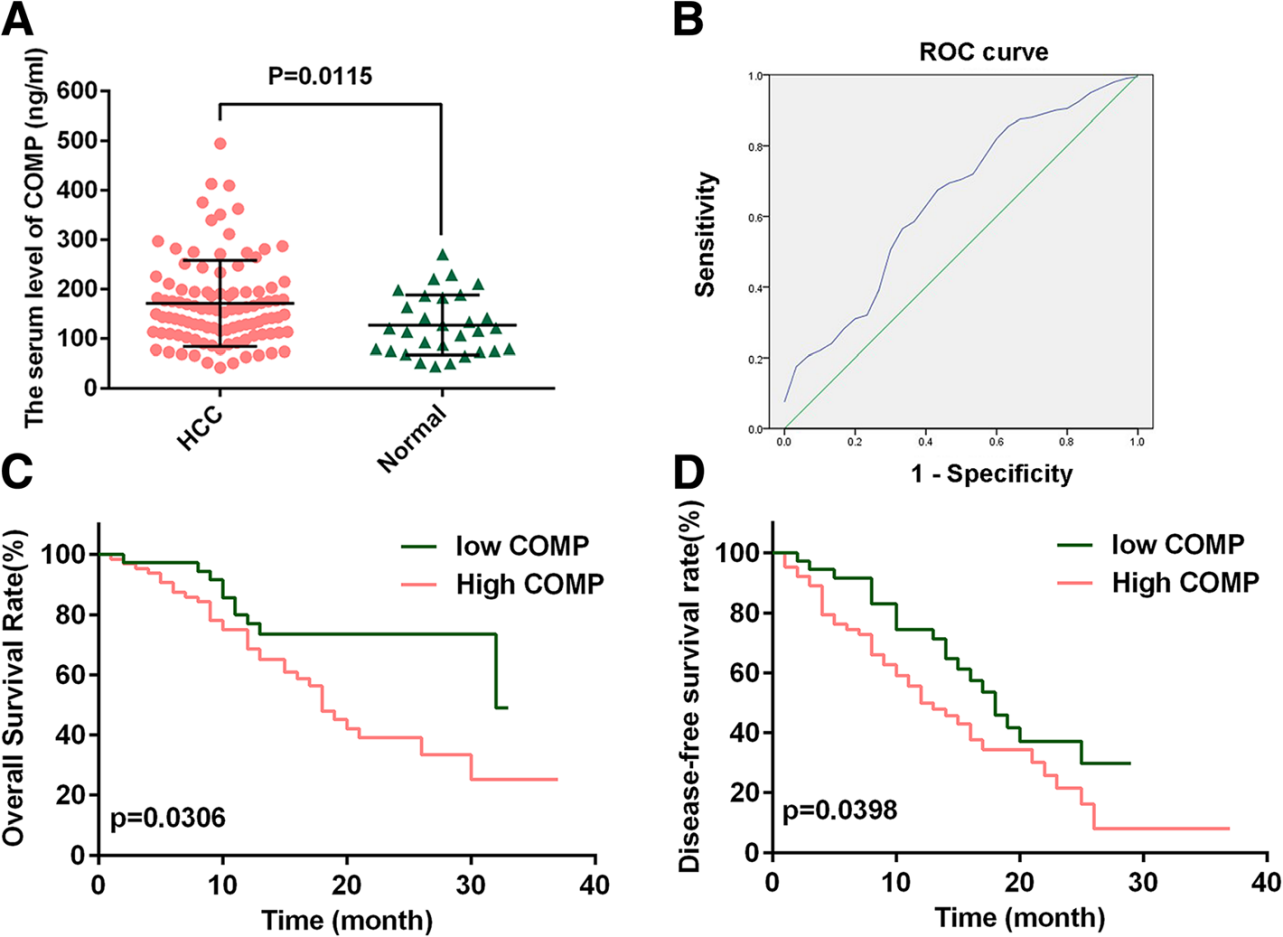
COMP level is increased in the serum of HCC patients. **a** ELISA analysis of COMP serum level in 100 HCC patients and 30 healthy controls. *P* = 0.0115 by t test versus normal controls. **b** ROC curve of serum COMP in 100 HCC patients and 30 normal controls. **c** and **d** The overall survival and disease-free survival of HCC patients with high or low level of COMP estimated using the Kaplan-Meier analysis and compared by the Log-rank test in the same set of patients

Our ELISA data showed that the serum content of COMP in the 100 HCC samples ranged from 41.9 ng/ml to 494.3 ng/ml with a median of 170.7 ng/ml. While, serum COMP level ranged from 44.2 ng/ml to 270.3 ng/ml with a median of 127.1 ng/ml in the 30 healthy controls, and a significant difference was observed between HCCs and healthy controls (*P* = 0.0115, Fig. 1a). According to the Youden index of the ROC curves (Fig. 1b), 127.7 ng/ml was determined as a optimal cutoff value for the diagnostic value of serum COMP in HCC. Results indicated that serum COMP was significantly upregulated in 64.0% (64/100) of HCC patients compared with the healthy controls (40.0%, 12/30, *P* = 0.019).

Next, a clinicopathological association analysis in the 100 HCCs found that the high-level of serum COMP was closely associated with poor clinicopathological features, including cirrhosis (*P* = 0.013), vascular invasion (*P* = 0.031), large tumor size (*P* = 0.020) and tumor recurrence (*P* = 0.043, Additional file 1: Table S1). Furthermore, Kaplan-Meier analysis showed that HCC patients with high serum COMP level had poorer overall survival rate (OS; Log-rank, 4.674; *P* = 0.0306, Fig. 1c) and disease-free survival rate (DFS; Log-rank, 4.227; *P* = 0.0398, Fig. 1d). Analyses using univariate Cox regression indicated that tumor size, cirrhosis, vascular invasion and high serum COMP level were all found to be significantly related with overall survival (*P* < 0.05, Table 1). Most importantly, multivariate Cox proportional hazard regression analysis found tumor size, vascular invasion, and serum COMP high-level to be independent prognostic factors for the overall survival of HCC patients (*P* < 0.05, Table 1). These results together underlined that elevated serum COMP level was closely correlated with HCC progression.

**Table 1:** Univariate and multivariate analysis of factors associated with overall survival in hepatocellular carcinoma patients

Features	Univariate Analysis			Multivariate Analysis		
	HR	95%Cl	*P*	HR	95%Cl	*P*
Age (≤50 versus > 50 years)	0.446	0.158–1.370	0.165			
Gender (Male versus Female)	2.069	0.612–6.996	0.242			
HBV (Negative versus Positive)	0.377	0.103–1.379	0.140			
Cirrhosis (Yes versus No)	3.878	1.096–13.721	***0.036***	2.750	0.980–7.716	0.055
AFP (> 400 versus ≤400 ng/mL)	2.235	0.667–7.487	0.192			
Vascular Invasion (Yes versus No)	3.524	1.038–11.962	***0.043***	3.408	1.159–10.026	***0.026***
Tumor Size (≤5 versus > 5 cm)	0.120	0.028–0.512	***0.004***	0.333	0.121–0.917	***0.033***
Tumor encapsulation (Complete versus No/incomplete)	0.363	0.121–1.092	0.071			
Differentiation (poor versus Well/moderate)	1.307	0.467–3.661	0.611			
TNM stage (II/III versus I)	1.546	0.456–5.235	0.484			
COMP (high versus low)	13.428	2.844–63.408	***0.001***	6.574	2.154–20.061	***0.001***

*HBV* hepatitis B virus, *AFP* alpha-fetoprotein, *TNM* tumor-node-metastasis, *HR* hazard ratio, *CI* confidence interval

Significant values (*P*<0.05) are in bold italic

### COMP shows a strong oncogenic function

**Fig. 2:**
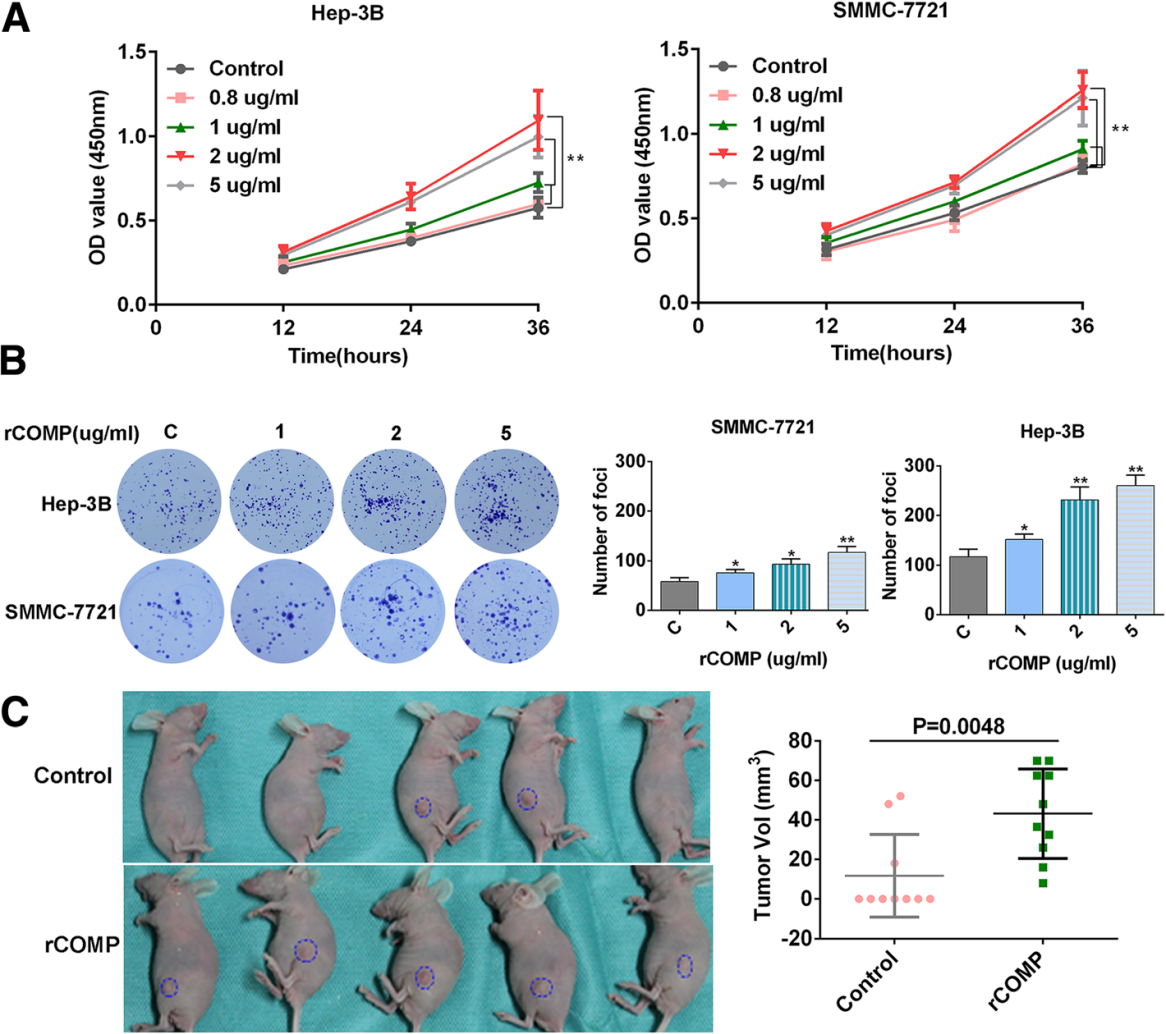
COMP promotes the proliferation of HCC cells in vitro and in vivo. **a** HCC cells were treated with rCOMP at various concentrations (as indicated) and the cell viability were determined using CCK8 assay. The untreated cells was considered to be the control group. n = three independent repeats. *P* < 0.05 by ANOVA versus control. **b** Plate colony formation assay was used to confirm the growth promotional effect of rCOMP at various concentrations and the colony foci from three independent experiments were calculated and compared. *P* < 0.05 by t test versus control. **c** SMMC-7721 cells with rCOMP or PBS were subcutaneously injected into the mouse flanks to establish subcutaneous tumor models (10 mice each group). After treatments, all mice were observed and the tumor volume was measured and compared. Representative images at × 200 magnification are shown. *P* < 0.05 by t test versus control. (*P < 0.05, **P < 0.01)

To explore the exact biological function of COMP in HCC, Hep-3B and SMMC-7721 cells were treated with different concentrations of rCOMP (0.8 μg/ml to 5 μg/ml), the proliferation activity of HCC cells was evaluated by CCK8. The presence of rCOMP significantly promoted the proliferative activity of HCC cells in a dose-dependent manner and the promoting effect of rCOMP peaked at 2 μg/ml (*P* < 0.05, Fig. 2a). We further examined the effect of rCOMP on the growth of HCC cells by using plate colony formation assay. Our data showed that the growth of rCOMP-treated cells was markedly enhanced in a dose-dependent manner, as compared with their respective controls (*P* < 0.05, Fig. 2b). To test whether rCOMP treatment also participate in HCC growth in vivo, the subcutaneous tumor model in nude mice was established by subcutaneously implanting SMMC-7721 cells with or without rCOMP pre-incubation. Notably, in the subcutaneous tumor model, it was observed that the tumor volume was significantly higher in the rCOMP treated group compared to control group (*P* = 0.0048, Fig. 2c).

### COMP enhances HCC cell invasion and tumor metastasis

**Fig. 3:**
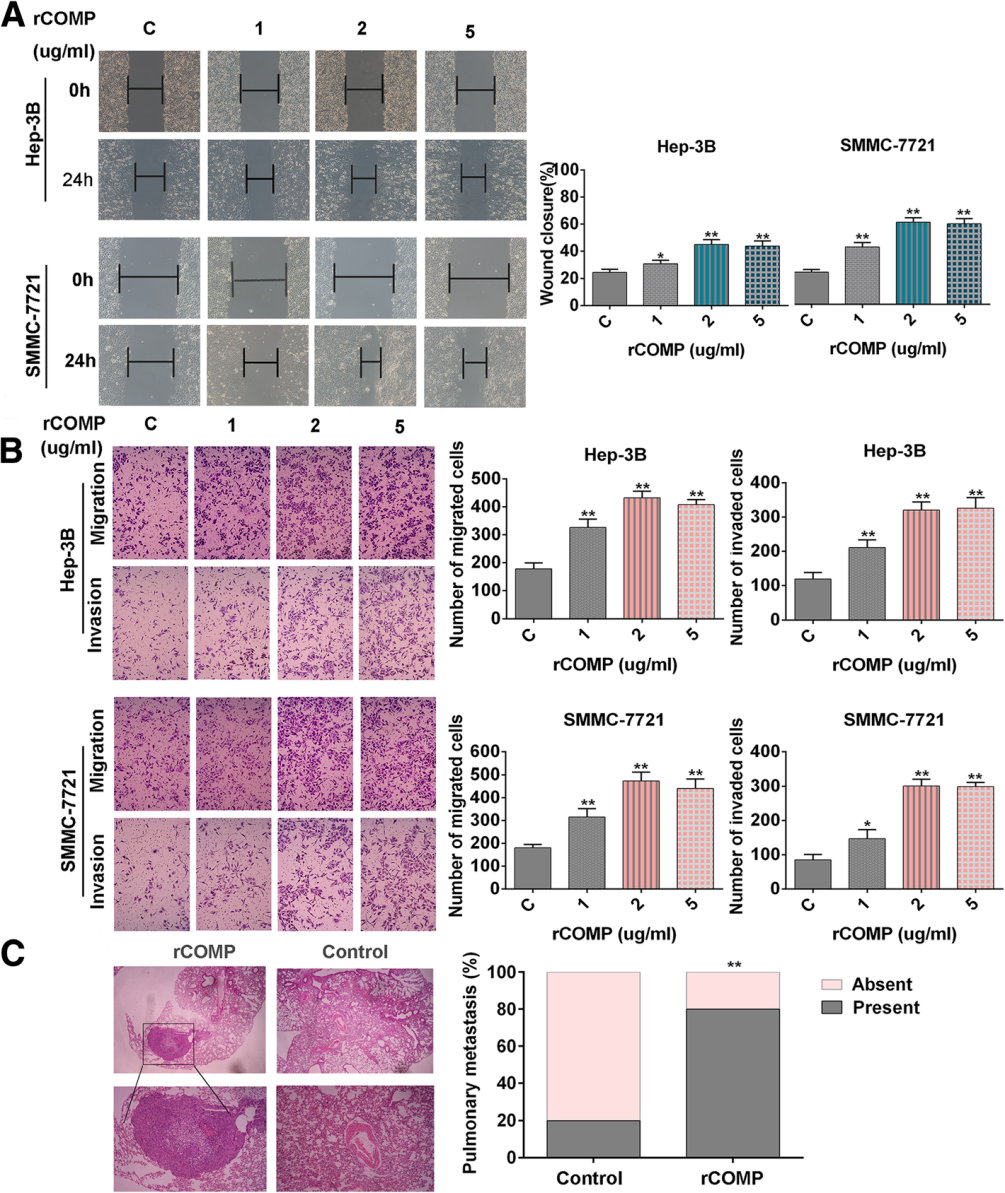
COMP promotes HCC cell migration and invasion in vitro and in vivo. **a** Hep-3B and SMMC-7721 cells incubated with various concentrations of rCOMP (as indicated) for 24 h were subjected to wound-healing assay. Representative images at × 400 magnification are shown. The wound closure (%) of HCC cells in each concentration of rCOMP was calculated. n = three independent repeats. *P* < 0.05 by t test versus control. **b** Transwell migration and invasion assays of HCC cells incubated with various concentrations of rCOMP (as indicated). The number of migrated or invaded cells was counted in five different fields. Representative images at *×* 200 magnification are shown. n = three independent repeats. *P* < 0.05 by t test versus control. **c** Invasive behavior of HCC cells was examined by injecting intravenously in the tail vein with SMMC-7721-rCOMP (*n* = 6) or SMMC-7721-PBS (Control, *n* = 6) cells; Lung metastasis were counted by H&E analysis. Representative images at *×* 200 magnification are shown. *P* < 0.05 by Pearson chi-square test versus control. (*P < 0.05, **P < 0.01)

Cancer progression is a multistep process that involves invasion of basement membrane by tumor cells and migration to points far from a given primary tumor mass, leading to metastasis [22]. Since COMP upregulation was significantly associated with HCC invasion, the role of COMP in tumor migration and invasion was further investigated. The wound healing assay showed that after incubation with rCOMP (1, 2, and 5 μg/ml) for 24 h, the migration distance of HCC cells were dose-dependently increased in comparison with untreated cells (*P* < 0.05, Fig. 3a). As expected, transwell migration assay confirmed the positive effect of rCOMP on migration potential of HCC cells after rCOMP incubation (*P* < 0.05, Fig. 3b). Next, as evident from Matrigel invasion assay, rCOMP treatment increased invasion of HCC cells through Matrigel in comparison with untreated cells (*P* < 0.05, Fig. 3b). In summary, rCOMP at a concentration of 2 μg/ml significantly induced the metastasis potential of HCC cells. The effect of COMP on HCC invasive behavior was examined by injecting intravenously in the tail vein with SMMC-7721-rCOMP or SMMC-7721-PBS cells to mimic tumor metastasis. Notably, a significantly larger number of pulmonary metastatic nodules were induced in the SMMC-7721-rCOMP group than those in the control group (*P* < 0.01, Fig. 3c). On the whole, these in vitro and in vivo results verified that COMP enhanced metastasis dissemination of HCC.

### COMP promotes HCC metastasis by enhancing EMT and MMP-2/9

**Fig. 4:**
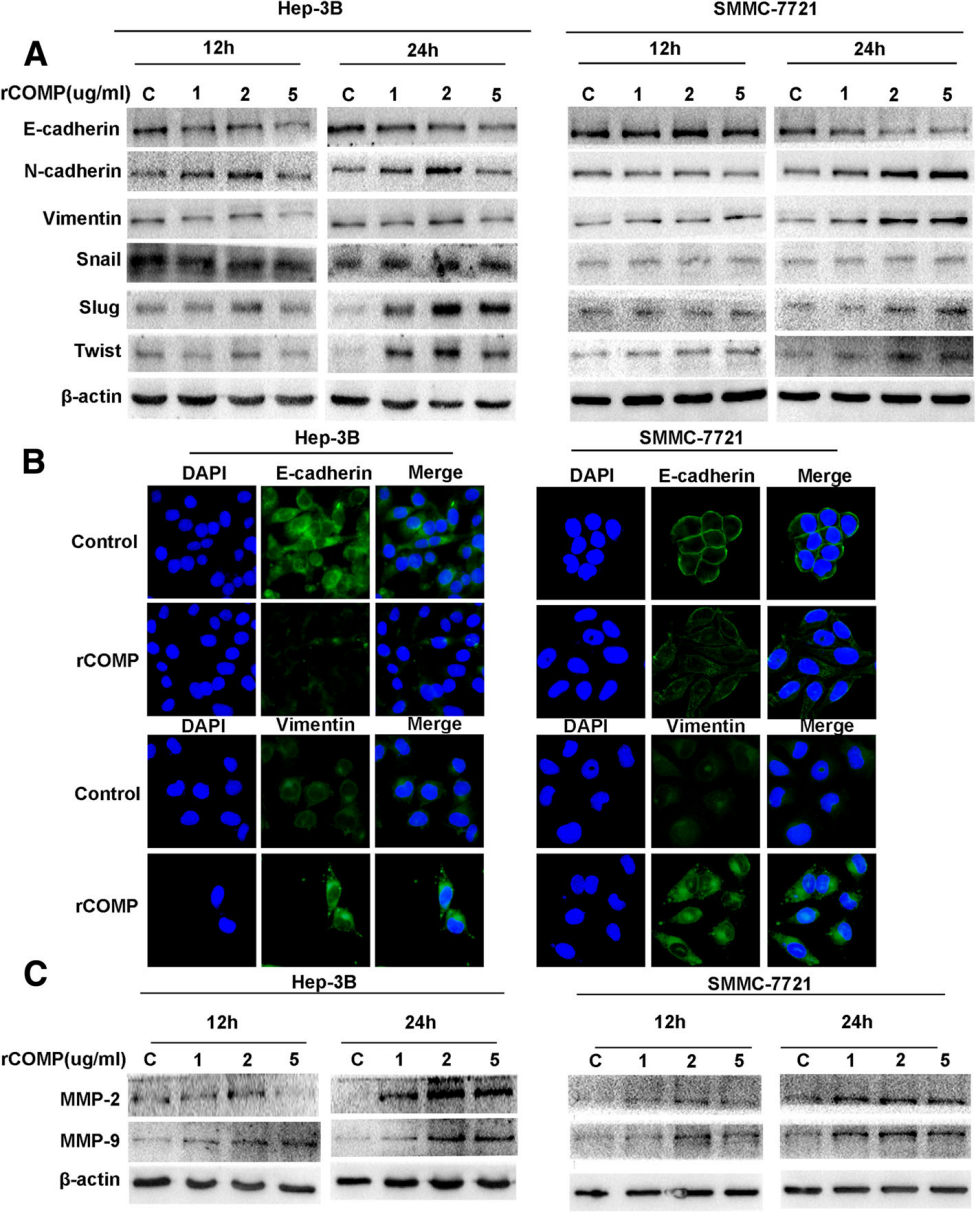
COMP facilitates EMT and MMP-2/9 expression in HCC cells. **a** The expression of EMT markers and transcription factors were determined via Western blot after treatment with various concentrations of rCOMP (as indicated) for 12 and 24 h. β-actin was used as a loading control. **b** The expression of E-cadherin (green) and vimentin (green) after treatment with rCOMP (2 μg /ml) were shown by immunofluorescence staining in both Hep-3B and SMMC-7721 cells. Representative images at *×* 400 magnification are shown. **c** The levels of MMP-2/9 in HCC cells after treatment with various concentrations of rCOMP (as indicated) for 12 and 24 h as detected by Western blot analysis. β-actin was used as a loading control. Western blot and IF analysis were independently repeated for three times with similar results

Tumor cell migration and invasion are often associated with reorganization of the actin cytoskeleton and a phenomenon called EMT. Malignant hepatocytes that are transdifferentiated to a mesenchymal phenotype during EMT process can escape from tumor mass by individual cell movement. To investigate whether COMP-mediated promotion of metastasis occurs via the EMT process, we analyzed the expression levels of EMT markers and EMT-related transcription factors after exposure to rCOMP. As showed in Fig. 4a, rCOMP dose-dependently decreased the expression of epithelial marker (E-cadherin) and increased the expression of mesenchymal markers (N-cadherin and Vimentin) in Hep-3B and SMMC-7721 cells at 24 h, compared with respective controls. Interestingly, alteration of E-cadherin expression was accompanied by an opposite parallel regulation of EMT regulators Slug and Twist rather than Snail, suggesting that Slug and Twist may play an important role in COMP-mediated EMT (Fig. 4a and Additional file 2: Figure S1). The changes of EMT phenotype after rCOMP treatment were further confirmed by immunofluorescence (Fig. 4b). We also detected the expression of several matrix metalloproteinases (MMPs), which were known to participate in ECM remodeling, an essential part of tumor metastasis. After rCOMP treatment, MMP-2 and MMP-9 levels were significantly upregulated at 24 h in a dose-dependent manner (Fig. 4c). These results were all typical of events that occur during EMT of tumor cells. In sum, these data further supported the efficacy of the rCOMP treatment in enhancing clonogenicity, migration and invasion of HCC cells.

### COMP activates the MEK/ERK and PI3K/AKT signaling pathways in HCC cells

**Fig. 5:**
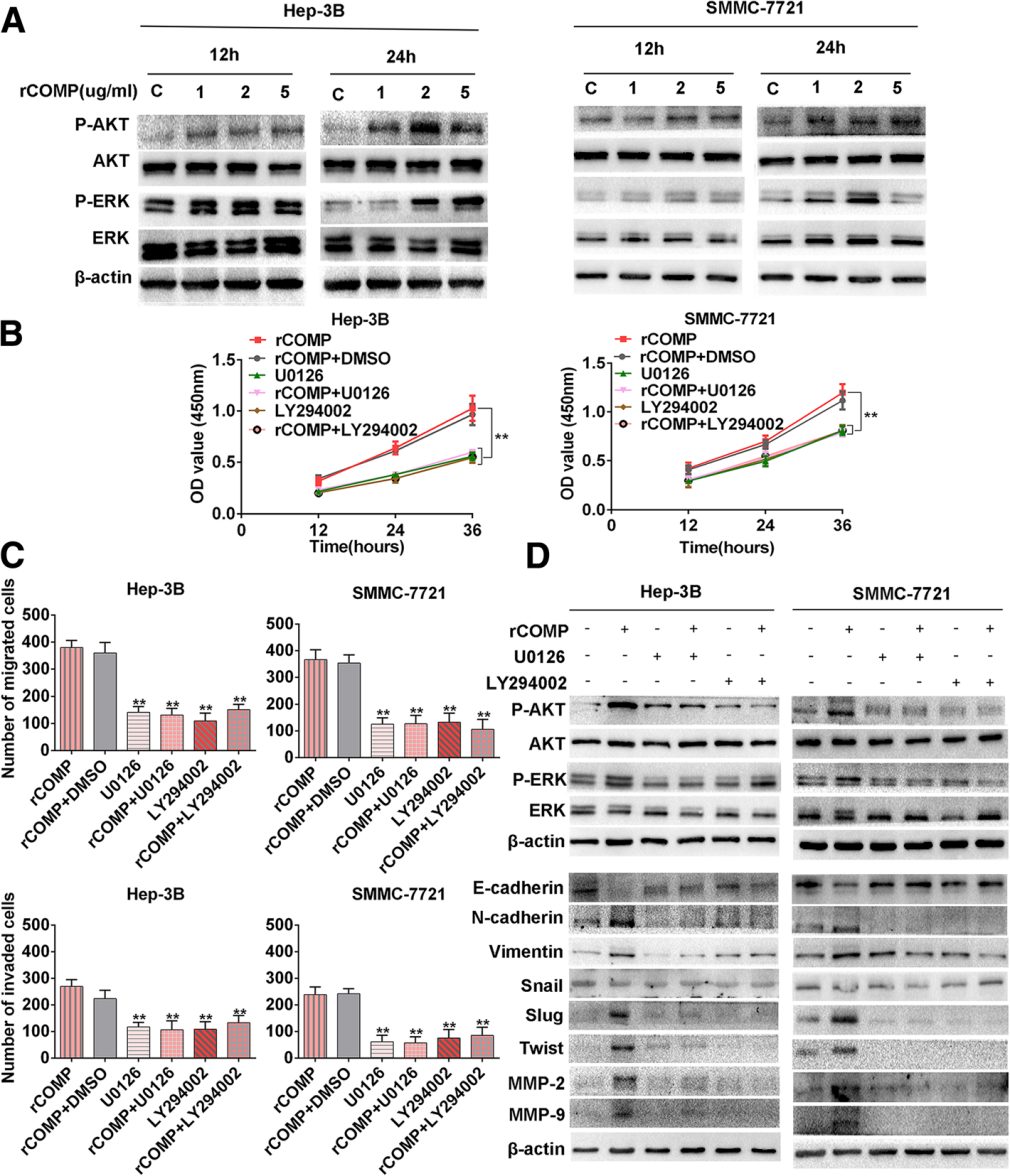
COMP activates MEK/ERK and PI3K/AKT pathway in HCC cells. **a** The levels of the indicated proteins in Hep-3B and SMMC-7721 after treatment with various concentrations of rCOMP (as indicated) as measured by Western blot. Total ERK and AKT were used as controls. **b** HCC cells were treated with rCOMP (2 μg /ml), rCOMP+DMSO, the MEK inhibitor U0126 (25 μM), rCOMP+U0126, PI3K inhibitor LY294002 (20 μM) and rCOMP+LY294002 for 12, 24 and 36 h*,* then the cell viability was evaluated using the CCK8 assay. The cell viability of every cell line with rCOMP+DMSO treatment was considered as control group. n = three independent repeats. *P* < 0.05 by ANOVA versus control. **c** HCC cells that were incubated with the indicated treatments were subjected to transwell migration and invasion assays. The number of migrated or invaded cells was counted in five different fields. n = three independent repeats. *P* < 0.05 by t test versus control. **d** The protein levels of the indicated factors after the indicated treatments were examined by Western blot. β-actin was used as a loading control. Western blot analysis was independently repeated for three times with similar results. (*P < 0.05, **P < 0.01)

Activation of MEK/ERK and PI3K/AKT has been shown to regulate cancer cell migration and invasion through distinct pathways by promoting the transcription activation of various transcription factors and MMPs-mediated matrix degradation [23, 24]. We examined whether rCOMP treatment affected MEK/ERK and PI3K/AKT activation to accelerate migration and invasion of HCC cells. The results showed that rCOMP treatment for 24 h significantly stimulated ERK and AKT phosphorylation in HCC cells in a dose-dependent manner without obvious changes of the total ERK and AKT expression levels, indicating the involvement of ERK and AKT phosphorylation in COMP-mediated promotion of migration and invasion potential of HCC cells (Fig. 5a). To confirm the role of MEK/ERK and PI3K/AKT pathways in proliferation and EMT process which regulated by COMP in HCC cells, the MEK inhibitor U0126 and PI3K inhibitor LY294002 were used. Interestingly, U0126 (25 μM) and LY294002 (20 μM) treatments in rCOMP-treated cells evidently inhibited the ability in proliferation than the rCOMP-treated controls, reversing changes caused by rCOMP (*P* < 0.05, Fig. 5b and Additional file 3: Figure S2A). Meanwhile, either U0126 or LY294002 treatment effectively suppressed migratory and invasive ability of rCOMP-treated cells (*P* < 0.05, Fig. 5c and Additional file 3: Figure S2B-C). Western blot results indicated that reduced E-cadherin expression in rCOMP-treated cells was recovered by U0126 or LY294002, whereas up-regulated mesenchymal markers (N-cadherin and Vimentin), MMP-2/9, Slug and Twist were reduced by U0126 or LY294002 treatments (Fig. 5d). Collectively, we concluded that rCOMP could induce HCC progression by regulating the activity MEK/ERK and PI3K/AKT pathways.

### CD36 receptor mediated COMP induced MEK/ERK and PI3K/AKT activation

**Fig. 6:**
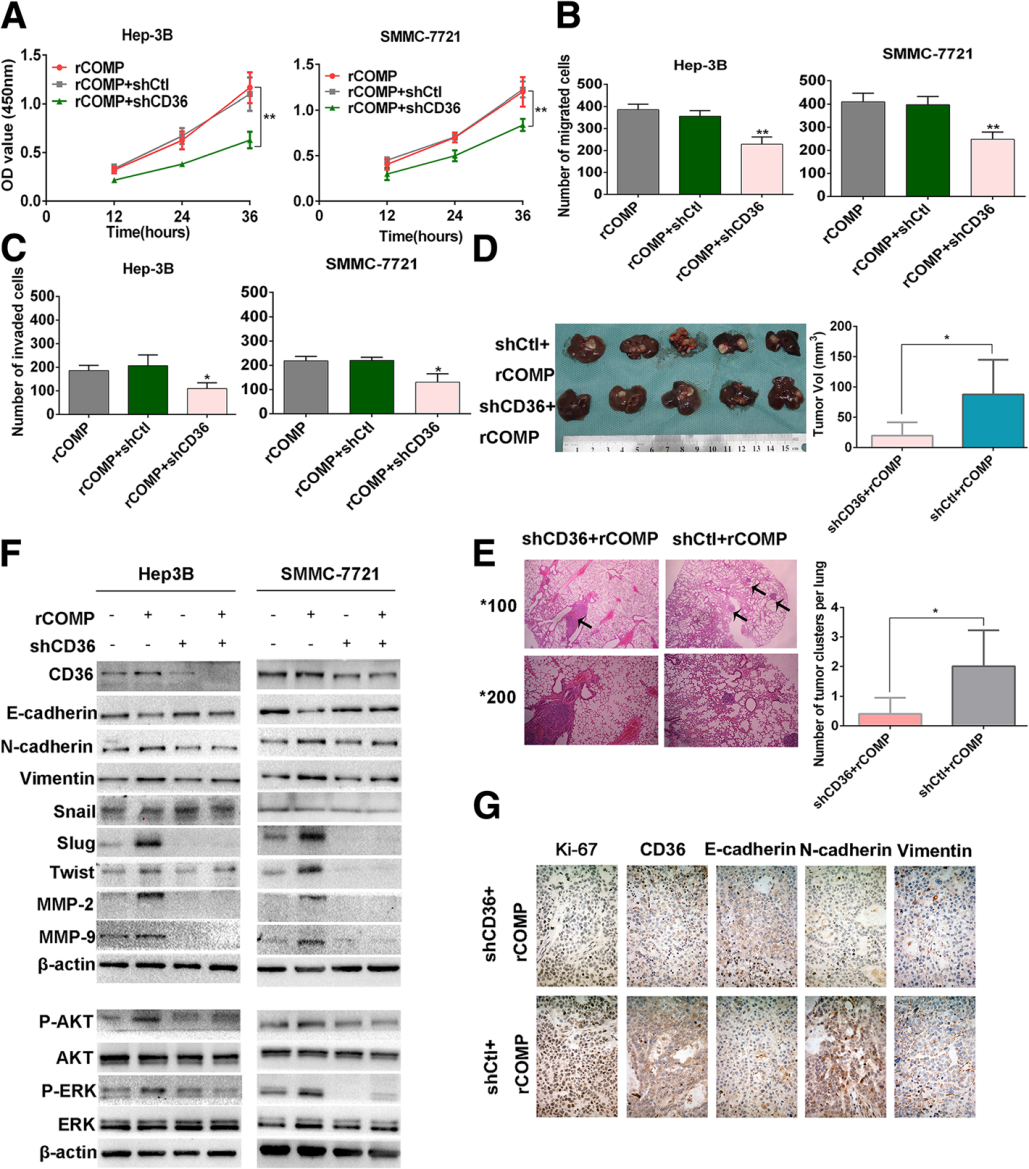
CD36 mediates COMP induced MEK/ERK and PI3K/AKT pathways activation. **a** Cell viability after CD36 knockdown by shRNA was determined using CCK8 assay. The cell viability of every cell line with rCOMP+shCtl treatment was considered as control group. n = three independent repeats. *P* < 0.05 by ANOVA versus control. **b** and **c** HCC cells after knockdown of CD36 were subjected with transwell migration and invasion assays. The number of migrated or invaded cells was counted in five different fields*.* n = three independent repeats. *P* < 0.05 by t test versus control. **d** Photomicrographs were taken for orthotopic primary liver tumors formed by shCD36 + rCOMP or shCtl+rCOMP (left). Tumor volumes from each group (*n* = 5) were measured (right). *P* < 0.05 by t test versus shCtl+rCOMP. **e** Representative H&E-stained sections of the lung tissues from the two groups were showed in the left. Magnification × 200. A total of 10 random visual fields were chosen from different lung sections of each group, and pulmonary foci were quantified as the average number across the 10 visual fields per group (right). *P* < 0.05 by t test versus shCtl+rCOMP. **f** The expression of the indicated proteins in HCC cells after CD36 knockdown by shRNA compared with controls in Hep-3B and SMMC-7721 cells. CD36 knockdown was confirmed by Western blot. β-actin was used as a loading control. Western blot analysis was independently repeated for three times with similar results. **g** The expression of Ki67, CD36, E-cadherin, N-cadherin and vimentin in xenograft tumors from different groups were analyzed by immunohistochemistry. Representative images at × 200 magnification are shown. (*P < 0.05, **P < 0.01)

Given the prior literatures about liver fibrosis suggesting that COMP induced fibrillary collagen-I deposition via CD36 receptor [4], we wonder if CD36 was related with COMP in HCC. Knockdown of CD36 in Hep-3B and SMMC-7721 cells notably attenuated the rCOMP-induced promotion of cell proliferation and metastasis (*P* < 0.05, Fig. 6a-c and Additional file 4: Figure S3A-C). In our in vitro analyses, we discovered the involvement and requirement of CD36 in biologic functions of COMP. Furthermore, we evaluated whether CD36 was involved in the process of COMP-induced HCC progression by establishing an orthotopic liver tumor model in nude mice. The results showed that CD36 knockdown attenuated the effect of rCOMP-induced promotion of tumor cell proliferation and lung metastasis (*P* < 0.05, Fig. 6d and e). More importantly, the reduction of E-cadherin due to rCOMP treatment in two HCC cells could be recovered by CD36 knockdown (Fig. 6f). Likewise, knockdown of CD36 abrogated the effect of rCOMP-induced upregulation of N-cadherin, Vimentin, Slug, Twist, MMP-2/9, P-ERK and P-AKT expression (Fig. 6f). These data demonstrated a mechanism by which COMP induced EMT by way of CD36/ERK and CD36/AKT pathway. The IHC analysis of liver tumor displayed higher level of E-cadherin and lower levels of N-cadherin and Vimentin and Ki-67 in the group of CD36 knockdown (Fig. 6g), which was consistent with the results in Fig. 6f. Together, these results presented that functional significance of CD36 was involved in the COMP induced tumor grwoth and metastasis of HCC. Taken together, these data suggested a crucial role for COMP in regulation of EMT through control of CD36-ERK/AKT-Slug/Twist axis in HCC cells.

### COMP is one of HSCs-derived factors that drives HCC progression

**Fig. 7:**
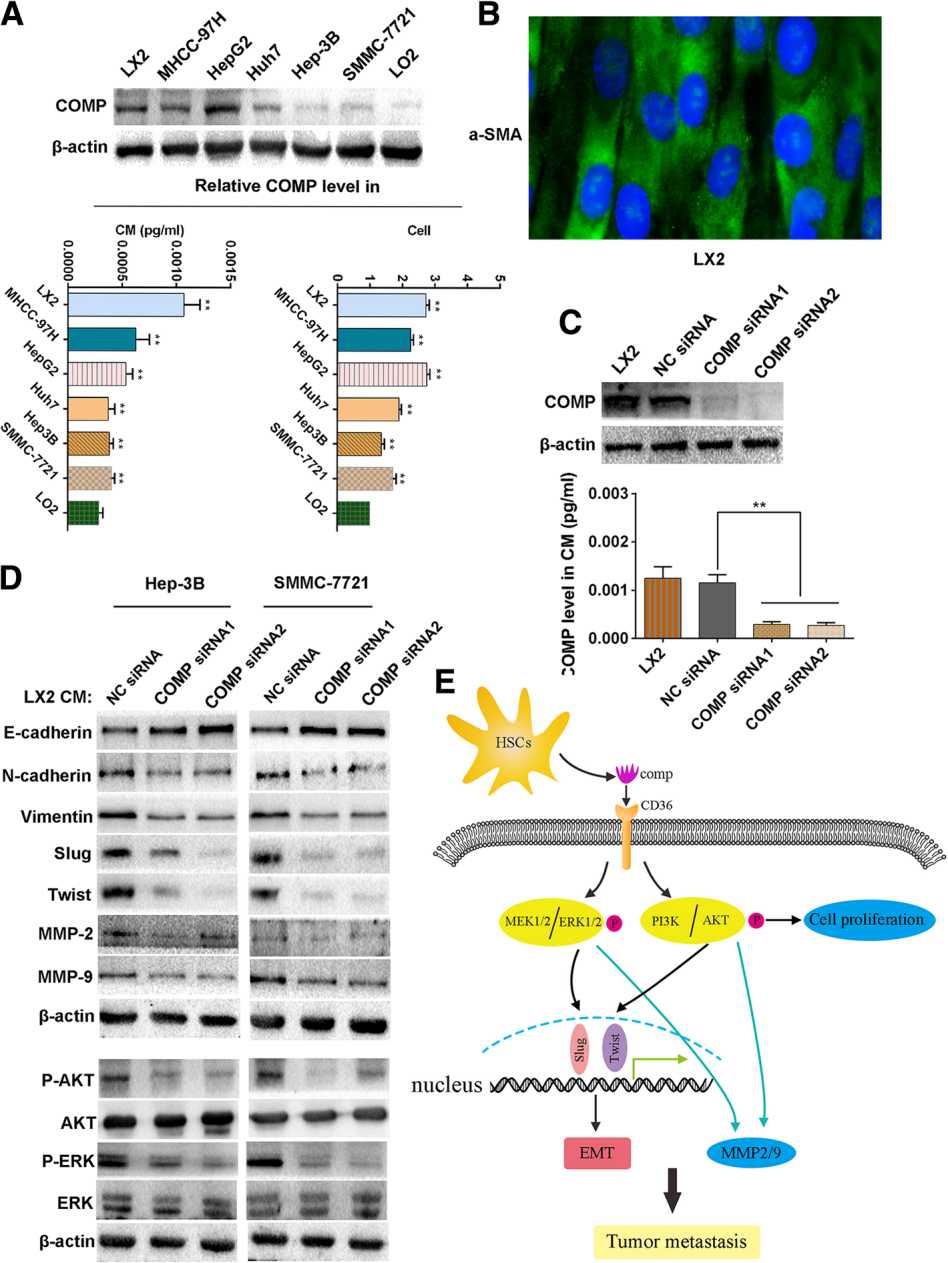
LX2 cells-derived COMP drives tumor progression. **a** COMP concentrations (detected by ELISA) in conditioned media (CM) and COMP expression (detected by Western blot) in 5 HCC cell lines and hepatocytes LO2 and activated hepatic stellate cell LX2. LO2 was used as a negative control. n = three independent repeats. *P* < 0.05 by t test versus LO2. **b** The marker of activated hepatic stellate cells α-SMA was confirmed using IF. Representative images at *×* 400 magnification are shown. **c** The level of COMP in the LX2 and CM was confirmed by Western blot and ELISA after knockdown by siRNAs. The NC siRNA was used as control. n = three independent repeats. *P* < 0.05 by t test versus control. **d** The expression of the indicated proteins in HCC cells after co-cultured with LX2 cells after knockdown of COMP were examined by Western blot. β-actin was used as a loading control. Western blot analysis was independently repeated for three times with similar results. **e** The proposed model by which HSCs-derived COMP promotes HCC progression by activating MEK/ERK and PI3K/AKT signaling pathway via a CD36-dependent manner. (*P < 0.05, **P < 0.01)

From clinical data, we concluded that COMP level was closely correlated with cirrhosis and HCC, therefore we designed experiments to detect whether the main source of COMP was from HSCs. The expression of COMP in activated hepatic stellate cell line LX2 and 5 HCC cell lines as well as one immortalized liver cell line LO2 were tested by Western blot analysis. The results showed that COMP was obviously highly expressed in LX2 cells (Fig. 7a). Besides, we also found that the level of COMP in cell culture supernatant as detected by ELISA was the highest in LX2 cells (*P* < 0.05, Fig. 7a), which was consistent with the findings of Western blot. These results suggested that COMP might be mainly secreted by activated hepatic stellate cells. Next, more experiments were performed to fully explore the biological significance of HSCs-derived COMP in HCC. Firstly, LX2 activation maker α-SMA was confirmed by IF (Fig. 7b). Knockdown of COMP by two different siRNAs in LX2 consistently inhibited the expression and secretion of COMP (*P* < 0.05, Fig. 7c). Conditioned medium (CM) of LX2 cells with or without COMP knockdown were cocultured with Hep-3B or SMMC-7721 cells for 24 h. These results indicated that knockdown of COMP significantly attenuated the tumor promoting effects of LX2 cells on HCC cells (*P* < 0.05, Additional file 5: Figure S4A-C). Then, we detected HCC cells with molecular markers of EMT. E-cadherin expression was obviously up-regulated, whereas mesenchymal markers such as N-cadherin, Vimentin and EMT regulators Slug and Twist were significantly down-regulated in HCC cells, which were treated with CM of COMP knockdown LX2 cells (Fig. 7d). Besides, the CM of COMP knockdown LX2 cells reduced MMP-2 and MMP-9 levels compared to the control (Fig. 7d). Moreover, the phosphorylation of ERK and AKT were significantly decreased in the CM of COMP knockdown LX2 treated HCC cells (Fig. 7d). These data indicated that COMP was one of HSCs derived factors and played an important role in controlling HCC cell proliferation and metastasis. In conclusion, HSCs-derived COMP promoted HCC progression by activating MEK/ERK and PI3K/AKT signaling pathway in a CD36-dependent manner (Fig. 7e).

## Discussion

The process of viral hepatitis-cirrhosis-HCC is the main epidemiological development path of HCC in world. Most (80%) of HCCs originate from severe liver fibrosis or cirrhosis [2]. Increased hepatic matrix stiffness results from the deposition and cross-linking of large amounts of matrix proteins, not only extensively occurs in most solid tumors but also promotes cell growth, motility, proliferation, metabolism and tumor metastasis [25–28]. HSCs, multifunctional hepatic stromal cells, differentiate into fibrogenic, hyperproliferative, contractile, and migrating myofibroblasts in chronic liver disease. In fibrosis and cirrhosis, these myofibroblastic HSCs are the culprits for many abnormal ECM deposits. Several publications revealed that COMP was involved with process of cirrhosis and HCC progression. However, the exact sources and functions of COMP still remain to be fully elucidated in HCC-related literature. In this regard, we demonstrated for the first time that COMP was mainly derived from activated HSCs and dose-dependently promoted HCC growth and metastasis. COMP induced CD36-dependent activation of MEK/ERK and PI3K/AKT, and a panel of tumor-promoting factors, including EMT makers, MMP-2/9, Slug and Twist, so as to promote its tumor-promoting effects. Our data illustrated a novel signal transduction pathway for metastatic growth of HCC (Fig. 7e).

In the present study, we identified that the level of COMP was frequently elevated in the serum of HCC patients. Patients with high level of serum COMP showed more unfavorable disease parameters such as higher incidence of vascular invasion and tumor recurrence. Additionally, HCC patients with high serum COMP level had a poorer prognosis than those with low serum COMP level. These results suggest that COMP may play important oncogenic roles in HCC progression which is consistent with previous report [5]. Cell proliferation and migration have been reported to involve various growth factors, which bind to their receptors on the cell surface to activate downstream signaling pathways, leading to cytoskeletal reorganization and stimulation of cellular motility machinery [29]. Here the oncogenic effects of COMP on HCC pathogenesis were directly demonstrated in the current study by both in vitro and in vivo functional assays. Our finding indicated that either exogenous COMP treatment or HSCs coculture stimulated malignant behaviors, such as proliferation, invasion and migration of HCC cells. In both subcutaneous xenografts and the tail vein injection model, rCOMP group generated bigger primary tumors and more lung metastatic foci, indicating that COMP enhanced aggressive and metastatic properties of HCC. Besides, COMP did not affect cell apoptosis of HCC cell lines (data not shown). To our knowledge, this is the first report that COMP acts as a driver of HCC proliferation and metastasis.

The acquisition of invasive capabilities includes degradation of the cell matrix and turnover of cell-cell adhesion junctions [30]. Down-regulation of E-cadherin is a significant hallmark of EMT. In our study, rCOMP treatment in HCC cells led to the up-regulation of Slug/Twist, N-cadherin and Vimentin, and repressed expression of E-cadherin and thereby triggered EMT. It has been demonstrated that MMP-2/9 can regulate the degradation of the extracellular matrix (ECM), which plays an important role in cancer metastasis [31]. Our study also observed that COMP advanced the expression level of MMP-2/9. Therefore, this study clearly demonstrates that COMP functions as a metastasis inducer in HCC through promoting EMT via regulation of Slug/Twist and inducting matrix degradation.

Both the MER/ERK and PI3K/AKT signaling pathway are involved in the regulation of tumor cell growth, metabolism, proliferation, as well as metastasis and are frequently proved to be active in many different types of cancer [32]. A recent report indicated that COMP could promote the process of liver fibrosis through MEK/ERK signaling pathway [4]. In our research, both the phosphorylation level of ERK and AKT were dramatically induced by rCOMP. EMT markers, MMP-2/9 and Slug/Twist are well-known downstream regulators of MEK/ERK and PI3K/AKT signaling pathways. Therefore, as expected, the expression of these proteins were suppressed when ERK or AKT pathway were inhibited. Besides, a crosstalk between AKT and ERK signaling pathways could be observed in the results, which was consistent with other studies [33, 34]. Taken together, our data confirmed the pro-proliferative and pro-invasive effects of COMP in HCC.

It has been shown that CD36 is up-regulated in human HCCs and involved in EMT [10]. Thus, we found that knockdown of CD36 attenuated rCOMP-induced proliferation, migration and invasion of HCC cells. Moreover, down-regulation of CD36 led to severe inhibition of rCOMP-induced tumor growth and lung metastasis of HCC in vivo. Therefore, we conclude that CD36 is essentially required for COMP/ERK and COMP/AKT induced HCC progression. To our knowledge, this is the first report that COMP connects with CD36 to stimulate HCC metastasis, in addition to tumor proliferation and growth. However, further studies will be necessary to expand our knowledge of the relationship between COMP and CD36.

In HCC, metastatic microenvironment is consisted of hepatoma cells, activated hepatic stellate cells, extracellular matrix, and their secreted or released various cytokines to regulate tumor metastasis. These cells and matrix components interact in the presence of various cytokines and participate in the process of hepatocellular carcinoma metastasis [35]. It has been increasingly recognized that activated HSCs act as important contributors to tumor progression [36]. Identifying the critical pathway involved in this crosstalk could potentially improve the efficiency of treatment. In this study, we detected the expression of COMP in activated HSCs LX2 and several HCC cells and hepatocytes LO2. The result indicated that the level of COMP in LX2 cells and its supernatants was the highest. These data suggested that COMP might be primarily secreted by HSCs. To support this hypothesis, we further established hepatocytes-HSCs crosstalk to analyzing the role of COMP in HCC microenvironment. Interestingly, the MEK/ERK and PI3K/AKT pathways were activated in HCC cells by coculture of activated HSCs and hepatoma cells, along with the upregulation of their downstream factors. Furthermore, above factors were deregulated in Hep-3B and SMMC-7721 cells respond to the coculture with LX2 after knockdown of COMP. Thus, the data suggest that COMP plays an important role in the dynamic interactions between cancer cells and activated HSCs in the progression of hepatocellular carcinoma.

## Conclusions

We uncovered a novel mechanism by which HSCs-derived COMP was frequently elevated in serum samples of HCC patients and played a very important role in HCC development and progression by activating ERK and AKT signaling pathways via a CD36-dependent manner. A better understanding of the oncogenic mechanisms of COMP in HCC may contribute to identify a promising biomarker in HCC diagnosis and a novel therapeutic strategy in HCC treatment.

## Additional files

Additional file 1: **Table S1**. Association between clinicopathological parameters and serum COMP level in primary hepatocellular carcinoma. (DOCX 17 kb)

Additional file 2: **Figure S1.** rCOMP treatment up-regulates the levels of Slug and Twist mRNA in HCC cells. The relative mRNA levels of Slug and Twist were up-regulated by rCOMP treatment both in Hep3B and SMMC-7721 cells. Each experiment was repeated at least three times. **P < 0.01 by t test versus control. (TIF 212 kb)

Additional file 3: **Figure S2**. COMP facilitates growth and metastasis of HCC cells via MEK/ERK and PI3K/AKT pathways. A) The plate colony formation assay was used to assess the growth of HCC cells with the indicated treatments and the number of foci from three independent experiments were calculated and compared. *P* < 0.05 by t test versus rCOMP+DMSO. B) Hep-3B and SMMC-7721 cells were treated with the indicated treatments for 24 h, the effect of rCOMP on cell migration was measured by wound-healing assay. The wound closure (%) of HCC cells in each concentration of rCOMP was calculated. Representative images at × 400 magnification are shown. n = three independent repeats, *P* < 0.05 by t test versus rCOMP+DMSO. C) The representative images of transwell migration and invasion assays. Original magnification × 200. Each experiment was carried out in triplicate wells and repeated at least three times. (*P < 0.05, **P < 0.01). (TIF 3192 kb)

Additional file 4: **Figure S3**. CD36 is required for the oncogenic function of COMP. A) The plate colony formation assay was used to assess the growth of HCC cells after knockdown of CD36 and the number of foci from three independent experiments were calculated and compared. *P* < 0.05 by t test versus rCOMP+shCtl. B) HCC cells after knockdown of CD36 were subjected to wound-healing assay. The wound closure (%) of HCC cells in each concentration of rCOMP was calculated. Representative images at × 400 magnification are shown. n = three independent repeats, *P* < 0.05 by t test versus rCOMP+shCtl. C) The representative images of transwell migration and invasion assays at × 200 magnification are shown. Each experiment of wound-healing assay and transwell assay was carried out in triplicate wells and repeated at least three times. (*P < 0.05, **P < 0.01). (TIF 2580 kb)

Additional file 5: **Figure S4**. LX2 cells-derived COMP promotes tumor progression. A) The plate colony formation assay was used to assess the growth of HCC cells after cocultured with LX2 cells and the number of foci from three independent experiments were calculated and compared. *P* < 0.05 by t test versus NC siRNA. B) HCC cells after cocultured with LX2 cells were subjected to wound-healing assay. The wound closure (%) of HCC cells was calculated. Representative images at × 400 magnification are shown. n = three independent repeats, *P* < 0.05 by t test versus NC siRNA. C) Transwell migration and invasion assays of HCC cells after cocultured with LX2 cells. The number of migrated or invaded cells was counted in five different fields. Representative images at × 200 magnification are shown. Each experiment of wound-healing assay and transwell assay was carried out in triplicate wells and repeated at least three times. *P* < 0.05 by t test versus NC siRNA. (*P < 0.05, **P < 0.01). (TIF 3501 kb)

## Abbreviations

CM: Conditioned medium
COMP: Cartilage oligomeric matrix protein
ELISA: Enzyme-linked immunosorbent assay
EMT: Epithelial to mesenchymal transition
HBV: Hepatitis B virus
HCC: Hepatocellular carcinoma
HSCs: Human hepatic stellate cells
IHC: Immunohistochemistry
MMP: Matrix metalloproteinase
rCOMP: recombinant human COMP protein
ROC: Receiver operating characteristic
