# Supplementary material for: HSCs-derived COMP drives hepatocellular carcinoma progression by activating MEK/ERK and PI3K/AKT signaling pathways
Source: J Exp Clin Cancer Res. 2018 Sep 19;37:231. doi: 10.1186/s13046-018-0908-y (PMC6146743; doi:10.1186/s13046-018-0908-y)
Supplement: Supplementary file 1 — Table S1. Association between clinicopathological parameters and serum COMP level in primary hepatocellular carcinoma. (DOCX 17 kb) [file 13046_2018_908_MOESM1_ESM.docx]

**Table S1.** Association between clinicopathological parameters and serum COMP level in primary hepatocellular carcinoma

| Features | n | COMP level | | P value |
| --- | --- | --- | --- | --- |
|  |  | Low (n=36) | High (n=64) |  |
| Age (Year) |  |  |  |  |
| >50 | 46 | 16 | 30 | 0.815 |
| ≤50 | 54 | 20 | 34 |  |
| Gender |  |  |  |  |
| Male | 78 | 28 | 50 | 0.968 |
| Female | 22 | 8 | 14 |  |
| HBV status |  |  |  |  |
| Positive | 71 | 22 | 49 | 0.102 |
| Negative | 29 | 14 | 15 |  |
| Cirrhosis |  |  |  |  |
| Yes | 73 | 21 | 52 | **0.013*** |
| No | 27 | 15 | 12 |  |
| AFP (ng/ml) |  |  |  |  |
| ≥400 | 64 | 20 | 44 | 0.187 |
| <400 | 36 | 16 | 20 |  |
| Vascular invasion |  |  |  |  |
| Yes | 36 | 8 | 28 | **0.031*** |
| No | 64 | 28 | 36 |  |
| Tumor size (cm) |  |  |  |  |
| ≤5 | 43 | 21 | 22 | **0.020*** |
| >5 | 57 | 15 | 42 |  |
| Tumor encapsulation |  |  |  |  |
| Complete | 41 | 15 | 26 | 0.919 |
| No/incomplete | 59 | 21 | 38 |  |
| Differentiation |  |  |  |  |
| Well/moderate | 59 | 25 | 34 | 0.111 |
| Poor | 41 | 11 | 30 |  |
| Recurrence/Metastasis  after surgery |  |  |  |  |
| Yes | 63 | 18 | 45 | **0.043*** |
| No | 37 | 18 | 19 |  |
| TNM stage |  |  |  |  |
| I | 40 | 15 | 25 | 0.799 |
| II+III | 60 | 21 | 39 |  |

HBV, hepatitis B virus; AFP, alpha-fetoprotein; TNM, tumor-node-metastasis.

*, P<0.05 was considered statistically significant.
