# Supplementary figures and images for: HSCs-derived COMP drives hepatocellular carcinoma progression by activating MEK/ERK and PI3K/AKT signaling pathways
Source: J Exp Clin Cancer Res. 2018 Sep 19;37:231. doi: 10.1186/s13046-018-0908-y (PMC6146743; doi:10.1186/s13046-018-0908-y)

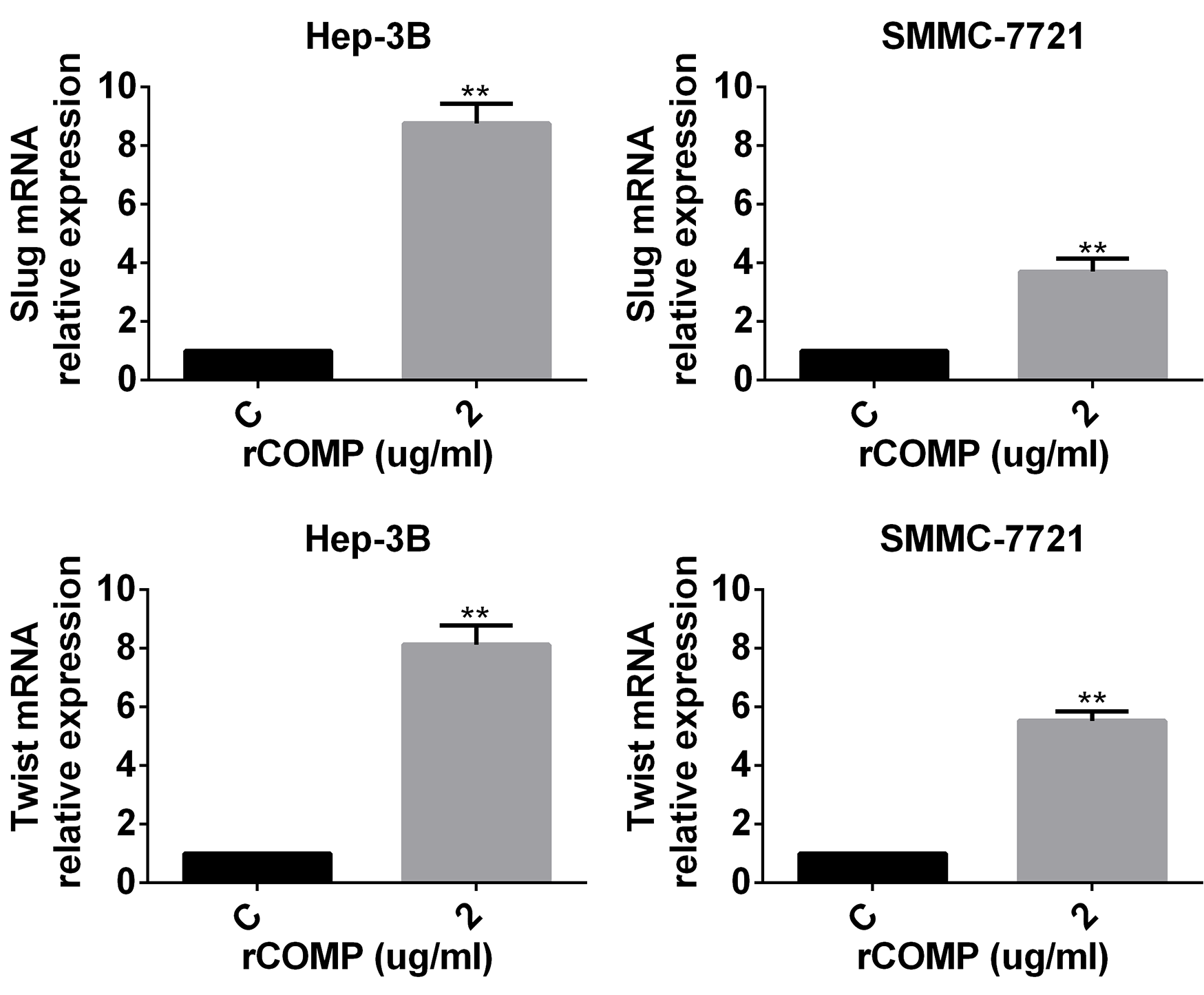

Supplement: Supplementary file 2 — Figure S1. rCOMP treatment up-regulates the levels of Slug and Twist mRNA in HCC cells. The relative mRNA levels of Slug and Twist were up-regulated by rCOMP treatment both in Hep3B and SMMC-7721 cells. Each experiment was repeated at least three times. **P < 0.01 by t test versus control. (TIF 212 kb) [file 13046_2018_908_MOESM2_ESM.tif]

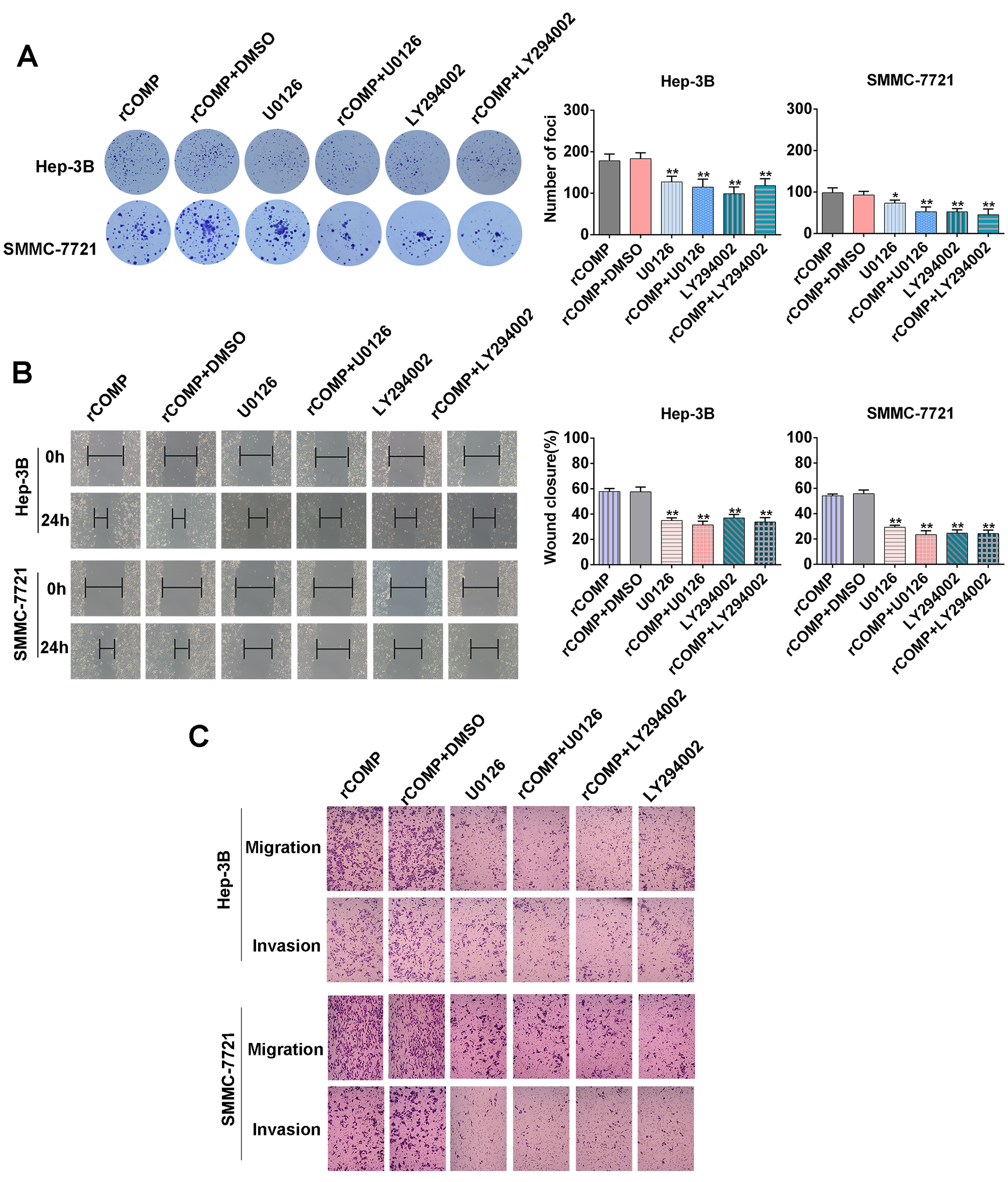

Supplement: Supplementary file 3 — Figure S2. COMP facilitates growth and metastasis of HCC cells via MEK/ERK and PI3K/AKT pathways. A) The plate colony formation assay was used to assess the growth of HCC cells with the indicated treatments and the number of foci from three independent experiments were calculated and compared. P < 0.05 by t test versus rCOMP+DMSO. B) Hep-3B and SMMC-7721 cells were treated with the indicated treatments for 24 h, the effect of rCOMP on cell migration was measured by wound-healing assay. The wound closure (%) of HCC cells in each concentration of rCOMP was calculated. Representative images at × 400 magnification are shown. n = three independent repeats, P < 0.05 by t test versus rCOMP+DMSO. C) The representative images of transwell migration and invasion assays. Original magnification × 200. Each experiment was carried out in triplicate wells and repeated at least three times. (*P < 0.05, **P < 0.01). (TIF 3192 kb) [file 13046_2018_908_MOESM3_ESM.tif]

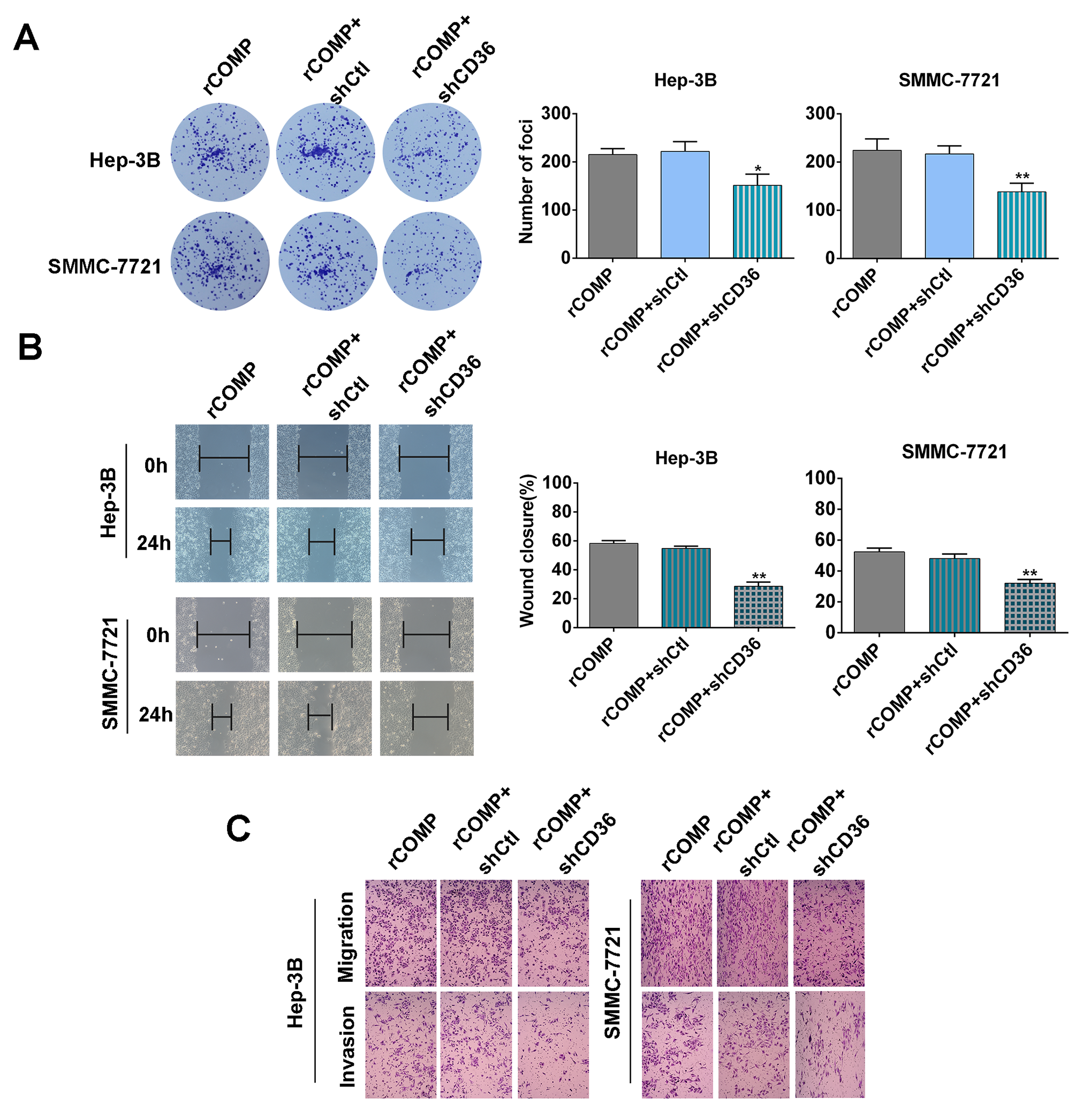

Supplement: Supplementary file 4 — Figure S3. CD36 is required for the oncogenic function of COMP. A) The plate colony formation assay was used to assess the growth of HCC cells after knockdown of CD36 and the number of foci from three independent experiments were calculated and compared. P < 0.05 by t test versus rCOMP+shCtl. B) HCC cells after knockdown of CD36 were subjected to wound-healing assay. The wound closure (%) of HCC cells in each concentration of rCOMP was calculated. Representative images at × 400 magnification are shown. n = three independent repeats, P < 0.05 by t test versus rCOMP+shCtl. C) The representative images of transwell migration and invasion assays at × 200 magnification are shown. Each experiment of wound-healing assay and transwell assay was carried out in triplicate wells and repeated at least three times. (*P < 0.05, **P < 0.01). (TIF 2580 kb) [file 13046_2018_908_MOESM4_ESM.tif]

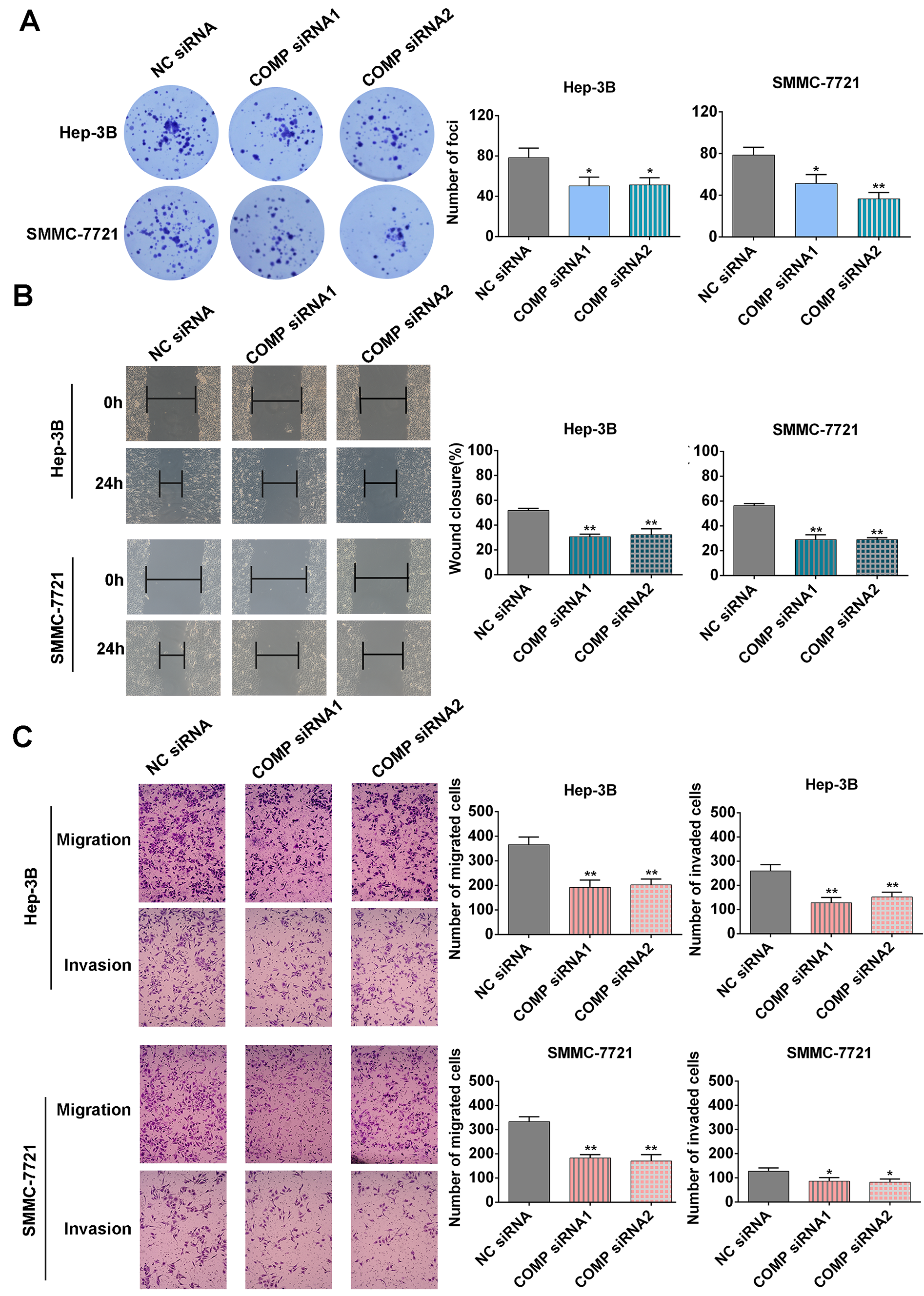

Supplement: Supplementary file 5 — Figure S4. LX2 cells-derived COMP promotes tumor progression. A) The plate colony formation assay was used to assess the growth of HCC cells after cocultured with LX2 cells and the number of foci from three independent experiments were calculated and compared. P < 0.05 by t test versus NC siRNA. B) HCC cells after cocultured with LX2 cells were subjected to wound-healing assay. The wound closure (%) of HCC cells was calculated. Representative images at × 400 magnification are shown. n = three independent repeats, P < 0.05 by t test versus NC siRNA. C) Transwell migration and invasion assays of HCC cells after cocultured with LX2 cells. The number of migrated or invaded cells was counted in five different fields. Representative images at × 200 magnification are shown. Each experiment of wound-healing assay and transwell assay was carried out in triplicate wells and repeated at least three times. P < 0.05 by t test versus NC siRNA. (*P < 0.05, **P < 0.01). (TIF 3501 kb) [file 13046_2018_908_MOESM5_ESM.tif]
